# Characterisation of cellular effects of *Burkholderia pseudomallei* cycle inhibiting factor (Cif)

**DOI:** 10.1242/bio.028225

**Published:** 2018-05-30

**Authors:** Mei Ying Ng, Yunn-Hwen Gan, Thilo Hagen

**Affiliations:** Department of Biochemistry, Yong Loo Lin School of Medicine, National University of Singapore, Singapore 117597, Singapore

**Keywords:** *Burkholderia pseudomallei*, Cycle inhibiting factor, Cullin RING E3 ubiquitin ligase, NF-κB

## Abstract

Cycle inhibiting factors (Cifs) are type III secretion system effectors produced by some Gram-negative pathogenic bacteria including *Burkholderia pseudomallei*. Through their deamidase activity, Cifs inhibit the activity of Cullin RING E3 ubiquitin ligases (CRL). CRL inhibition induces the accumulation of cell cycle inhibitors p21 and p27, thereby leading to host cell cycle arrest. However, whether Cif exerts additional effects on host cells that are important in bacterial pathogenesis is currently poorly understood. In this study, we found that Cif exerts a bimodal effect on NF-κB signalling. Cif increases basal NF-κB activity. This effect is dependent on Cif-mediated activation of ERK MAPK. On the other hand, Cif inhibits NF-κB activation by TNFα and *Burkholderia thailandensis* infection. This inhibitory effect on NF-κB activity is partially mediated by Cif-dependent inhibition of CRLs. We also found that Cif only has a modest effect in stimulating the intracellular replication of the *B. pseudomallei* surrogate, *B. thailandensis.* The observed Cif-dependent stimulation of *B. thailandensis* intracellular replication was not, or was only partially, due to CRL inhibition. Furthermore, the increased *B. thailandensis* replication induced by Cif was independent of ERK MAPK activation. Our findings suggest that Cif likely exerts additional cellular effects through novel targets.

## Abstract (precis)

**Summary:** Cycle inhibiting factor (Cif) is a *Burkholderia pseudomallei* virulence factor and is shown to exert both Cullin RING E3 ligase dependent and independent effects on host cells.

## INTRODUCTION

Deamidases are an important class of bacterial virulence factors capable of modulating normal host cell functions to benefit bacterial survival. Three classes of bacterial deamidases have been identified and characterised, including cytotoxic necrotising factors (CNFs), *Pasteurella Multocida* Toxin (PMT) and cycle inhibiting factors (Cifs) (Schmidt et al., 1997; Joachim et al., 2009; Cui et al., 2010). Cif was initially discovered in enteropathogenic *Escherichia coli* (EPEC) and enterohemorrhagic *E. coli* (EHEC), where it is injected into the infected cells via the type III secretion system (T3SS). Subsequently, Cif was also found to be present in four other pathogenic or symbiotic bacteria: *Yersinia pseudotuberculosis*, *Photorhabdus luminescens*, *Photorhabdus asymbiotica* and *Burkholderia pseudomallei* (Jubelin et al., 2009).

The *B. pseudomallei* Cif protein, which is also known as CHBP (Cif homolog in *B. pseudomallei*), was found to also be secreted into infected cells via the Burkholderia secretion apparatus (Bsa) T3SS (Pumirat et al., 2014). Cif is not present in the non-pathogenic *Burkholderia thailandensis* (Yao et al., 2009; Pumirat et al., 2014). CHBP is also not present in all *B. pseudomallei* strains. A recent analysis of 43 *B. pseudomallei* strains with known genome sequence found that Cif is present in 33 genomic sequences (Pumirat et al., 2014). Of note, a *B. pseudomallei* Cif insertion mutant showed a significant reduction in cytotoxicity compared to the wild-type strain (Pumirat et al., 2014).

The main targets of Cif in host cells appear to be Cullin RING E3 ubiquitin ligases (CRLs) (Cui et al., 2010; Jubelin et al., 2010). CRLs are a large family of multi-subunit modular E3 ligases that control the stability of hundreds of cellular proteins (Petroski and Deshaies, 2005; Bosu and Kipreos, 2008). All CRLs contain one of seven Cullin scaffold proteins, as well as a RING domain protein (Rbx1 or Rbx2) and one of several hundreds of substrate receptor proteins. The Cullin scaffold subunit is normally covalently modified with the ubiquitin-like protein NEDD8. The modification of Cullins with NEDD8 (known as neddylation) results in the activation of CRLs. Cif has been shown to catalyse the deamidation of glutamine 40 in NEDD8 to glutamate, thereby leading to inhibition of CRL-catalysed polyubiquitin chain formation. Inhibition of CRL activity by Cif results in the stabilisation of CRL substrates, including the cell cycle inhibitors p21 and p27. Indeed, accumulation of p21 and p27 and induction of cell cycle arrest were the initially noted consequences of Cif expression in mammalian cells and gave the virulence factor its name (cycle inhibiting factor) (Samba-Louaka et al., 2008; Cui et al., 2010; Morikawa et al., 2010). It is thought that cell cycle inhibition is advantageous for bacteria as it slows down the renewal of the infected epithelium and lengthens the time window during which bacteria can replicate. By inhibiting cell cycle progression, Cif may promote bacterial colonisation in the epithelial cell layer.

A recent study by McCormack et al. (2015) identified a novel mechanism through which Cif promotes virulence during infection with *Y. pseudotuberculosis*. The authors showed that Cif inhibits perforin-2 activity. Perforin-2 is a mediator of the innate immune response of host cells and induces bacterial lysis. It was found that upon *Y. pseudotuberculosis* infection, perforin-2 becomes monoubiquitinated via a CRL, resulting in activation of perforin-2. By mediating NEDD8 deamidation and inhibiting CRL activity, Cif was shown to block the perforin-2-dependent innate immune response.

The described mechanism in *Y. pseudotuberculosis* infection is unlikely to be of major importance during *B. pseudomallei* infection. *B. pseudomallei* likely avoids bactericidal activity of perforin-2 by being able to readily escape from endosomes into the host cytosol (Stevens et al., 2002). Hence, Cif in *B. pseudomallei* may exert effects via other targets. The goal of the current study was to identify novel mechanisms through which Cif may regulate host cell function and promote *B. pseudomallei* intracellular replication. We found that Cif exhibits a number of cellular effects, including the inhibition of NF-κB signalling and the activation of ERK MAPK signalling. Our results suggest that, besides CRL inhibition, Cif is likely to have additional cellular targets.

## RESULTS

### Cif stabilises CRL substrates in a manner dependent on its catalytic activity

**Fig. 1.**
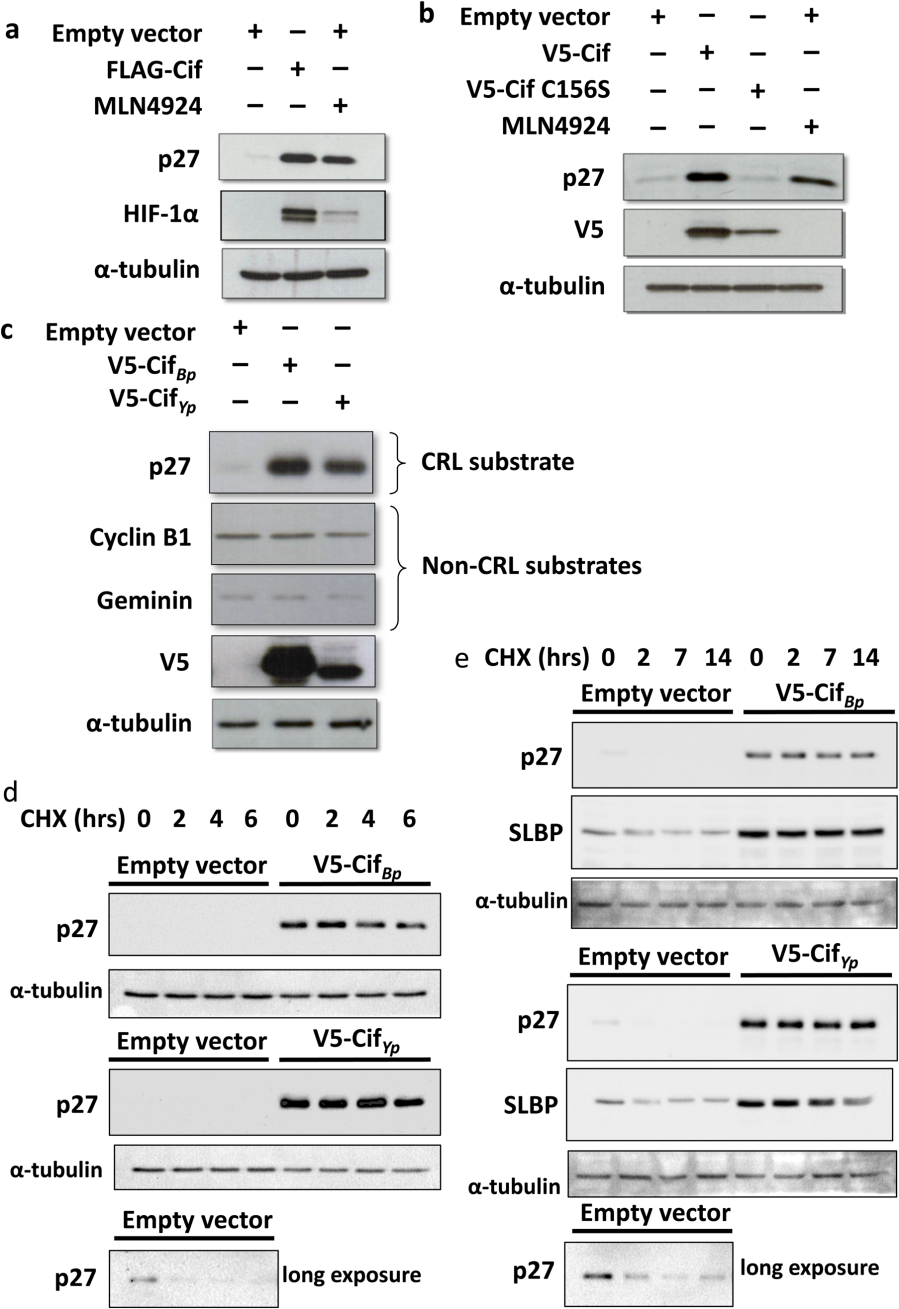
**Cif-mediated CRL substrate stabilisation.** (A,B) HEK293T cells were transfected with FLAG-tagged Cif, V5-tagged Cif or Cif C156S for 48 h and treated with 1 μM MLN4924 for the last 24 h. Cell lysates were resolved using SDS-PAGE and the cellular abundance of CRL substrates was analysed using Western blotting. (C) Cells were transfected with V5-tagged Cif*_Bp_* or Cif*_Yp_* for 48 h before cell lysis. (D,E) Cells were transfected with V5-tagged Cif*_Bp_* or Cif*_Yp_* for 48 h before chase with 40 μM cycloheximide for the indicated times, followed by cell lysis and Western blotting.

It is well known that Cif catalyses the deamidation of NEDD8, thereby causing the stabilisation of CRL substrates. We initially validated the effect of Cif on the cellular abundance of CRL substrates. This was done by transient transfection of Cif plasmid, amplified from *B. pseudomallei* strain K96243 (Boh et al., 2011), in HEK293T cells. As a positive control, the NEDD8 E1-activating enzyme inhibitor MLN4924, which blocks all CRLs, was used (Soucy et al., 2009). Cif markedly induced the accumulation of CRL substrates p27 and HIF-1α compared to cells transfected with empty vector (Fig. 1A). Similarly, upon treatment with MLN4924, an increase in p27 and HIF-1α protein levels was detected, but notably to a lesser extent compared to the effect of Cif. Thus, Cif transfection proved to be more potent than MLN4924 in stabilising CRL substrates p27 and HIF-1α.

To further validate that the effect of Cif on CRL substrate stabilisation is dependent on its deamidation activity, a catalytically inactive Cif mutant, as previously described by Yao et al. (2009), was constructed. The critical cysteine residue at position 156 in the catalytic domain was mutated to serine via site-directed mutagenesis. Indeed, when this catalytically inactive Cif mutant (Cif C156S) was expressed in cells, no significant change in the cellular abundance of p27 was observed compared to control cells. In contrast, wild-type Cif was capable of inducing the accumulation of p27 (Fig. 1B). We consistently observed that the Cif C156S mutant exhibited lower expression levels compared to the wild-type protein. Nevertheless, the absence of any effect of the catalytically inactive Cif mutant strongly suggests that the catalytic activity of Cif is required for the inhibition of CRL function.

We also compared the activity of Cif from *B. pseudomallei* (Cif*_Bp_*) with another Cif homolog from *Yersinia pseudotuberculosis* (Cif_*Yp*_). As expected, when Cif*_Yp_* was transfected into cells, an accumulation of the CRL substrate p27 was observed (Fig. 1C). Indeed, Cif*_Bp_* and Cif*_Yp_* increased the stability of the CRL substrates p27 and stem-loop binding protein (SLBP) similarly, as measured by performing cycloheximide chase (Fig. 1D,E). Of note, although the stabilisation of p27 with Cif*_Yp_* was comparable to Cif*_Bp_*, the expression level of Cif*_Yp_* was lower than that of Cif*_Bp_* (Fig. 1C). Therefore, considering this difference in Cif expression, the effect of Cif*_Yp_* on the stabilisation of p27 appears to be more potent compared to Cif*_Bp_*. In addition, when the steady-state levels of ubiquitin-proteasome substrates including cyclin B1 and geminin, whose ubiquitination is not mediated by CRLs, were measured, no significant changes were observed. Thus, similar to Cif*_Bp_*, Cif*_Yp_* appears to specifically inhibit CRL function via NEDD8 deamidaton.

### Cif inhibits *B. thailandensis*-induced NF-κB activity but stimulates basal NF-κB activity

**Fig. 2.**
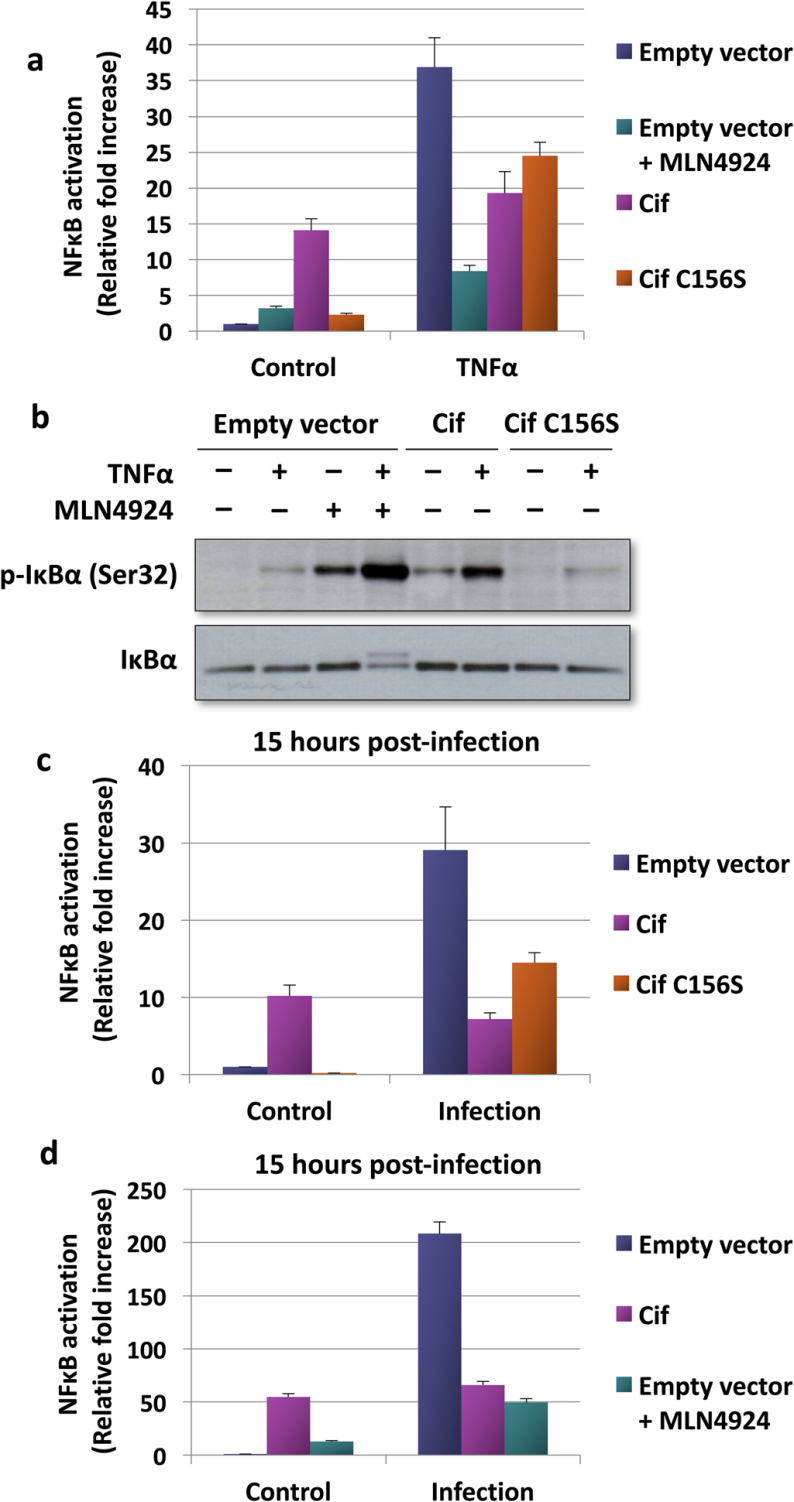
**Effect of Cif on NF-κB activity upon stimulation with TNFα or infection with *B. thailandensis*.** (A) Cells transfected with the indicated expression plasmids and an IL8 luciferase reporter plasmid were treated with 30 ng/ml TNFα and 1 μM MLN4924 for 8 h. NF-κB activation was then measured and fold change was presented as mean±s.d. The results shown are representative of two independent experiments. (B) Cell lysates from the reporter assay described in Fig. 3A were resolved using SDS-PAGE and the cellular protein level of phosphorylated IκBα was determined using Western blotting. (C,D) Cells transfected with the indicated expression plasmids and an IL8 luciferase reporter plasmid were pre-treated with 1 μM MLN4924 for 1 h prior to infection with *B. thailandensis* for 15 h as described in the Materials and Methods. NF-κB activation was then measured at the stipulated time and fold change was presented as mean±s.d. The results shown are representative of two independent experiments.

Numerous studies have reported that bacterial effectors secreted via the T3SS modulate host inflammatory signalling (Mota and Cornelis, 2005). Therefore, one hypothesis for the mechanism of Cif to promote *B. pseudomallei* virulence and intracellular replication is through suppression of host innate immune response. To test this, we used the *B. pseudomallei* surrogate *B. thailandensis* and investigated the role of Cif in the regulation of NF-κB signalling. To this end, an IL8 luciferase reporter assay system was used to measure NF-κB transcriptional activity. To examine the effect of Cif on NF-κB activity, cells were co-transfected with the IL8 luciferase reporter plasmid and wild-type or catalytically inactive Cif. We initially used TNFα to stimulate NF-κB activity. Cif indeed inhibited TNFα-induced NF-κB activity (Fig. 2A). The CRL inhibitor MLN4924 also inhibited TNFα-induced NF-κB activation, suggesting that the effect of Cif may be due to CRL inhibition. On the other hand, the catalytically inactive Cif mutant surprisingly also inhibited TNFα-dependent NF-κB activity. This effect is likely due to a CRL-independent mechanism.

**Fig. 3.**
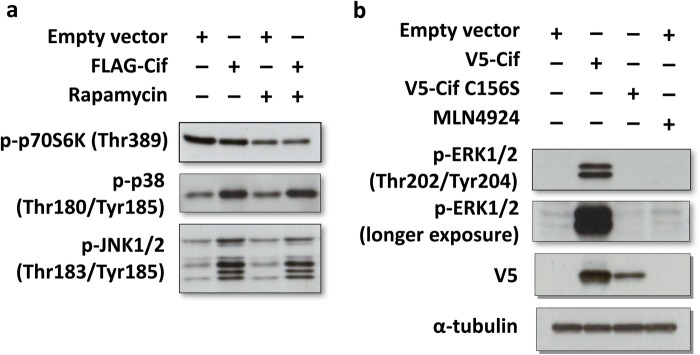
**Cif activates ERK MAPK.** (A,B) To measure the effect of Cif on cellular signalling, cells were transfected as indicated, followed by Western blotting of cell lysates with the indicated phospho-specific antibodies. The cells were treated with mTOR inhibitor rapamycin (20 nM) for 33 h or 1 μM MLN4924 for 24 h where indicated.

The NF-κB dimer p50/p65 is normally sequestered in the cytosol by IκBα. As a result, the translocation of the NF-κB dimers into the nucleus is prevented. TNFα and various other stimuli induce the phosphorylation and subsequent CRL-dependent degradation of IκBα, leading to NF-κB activation. As shown in Fig. 2B, TNFα treatment indeed caused an increase in IκBα phosphorylation. However, TNFα stimulation did not cause a decrease in the total IκBα protein level. This suggests that only a small proportion of the cellular IκBα pool was phosphorylated and degraded upon TNFα stimulation. Importantly, treatment with MLN4924 and transfection of wild-type but not C156S mutant Cif resulted in an increase in the phosphorylated IκBα level. This suggests that similar to MLN4924, Cif functions by inhibiting the CRL-dependent degradation of phosphorylated IκBα. Transfection of mutant Cif resulted in only a very small increase in IκBα degradation, consistent with a CRL independent mechanism of catalytically inactive Cif.

Of note, the NF-κB inhibition mediated by MLN4924 was much greater compared to wild-type Cif (Fig. 2A). This was unexpected, since Cif was shown to inhibit CRL more potently than MLN4924 (Fig. 1). Furthermore, we found that the catalytically inactive Cif mutant also inhibited TNFα-induced NF-κB activation to a similar degree compared to wild-type Cif (Fig. 2A). We noted that under basal conditions, wild-type Cif markedly stimulated NF-κB activity (Fig. 2A). In contrast, the catalytically inactive Cif mutant was without effect on the basal NF-κB activity. Thus, wild-type Cif has two opposing effects on NF-κB signalling: it increases basal NF-κB activity and decreases cytokine-induced NF-κB activation. This likely explains why wild-type Cif inhibits TNFα-induced NF-κB activation to a lesser degree compared to MLN4924. Only wild-type but not catalytically inactive Cif increased basal NF-κB activity. This result also explains why wild-type Cif does not inhibit TNFα-induced NF-κB activation to a greater degree compared to the Cif mutant.

We next investigated the effect of Cif on NF-κB activity in *B. thailandensis*-infected cells. To this end, cells were co-transfected with the IL8 luciferase reporter and the wild-type or catalytically inactive Cif mutant plasmids and then infected with *B. thailandensis* for 15 h. NF-κB activation at 15 h post-infection was then measured. As shown in Fig. 2C-D, Cif markedly inhibited *B. thailandensis*-induced NF-κB activation. Compared to wild-type Cif, the catalytic inactive Cif mutant inhibited *B. thailandensis*-dependent NF-κB activation to a lesser extent (Fig. 2C). When cells were treated with MLN4924, *B. thailandensis*-induced NF-κB activation was also dramatically inhibited (Fig. 2D), suggesting an involvement of CRL inhibition.

In conclusion, Cif exerts an activating effect on the basal NF-κB activity. Simultaneously, Cif inhibits both TNFα- and *B. thailandensis*-induced NF-κB activation, at least partially by inhibiting the CRL-dependent degradation of phosphorylated IκBα.

### Cif activates MAPK intracellular signalling pathways but not the mTOR pathway

Cif may also exert effects on host cells through the regulation of other intracellular signalling pathways. One such pathway is the mTORC1 pathway, which regulates autophagy. It is well known that autophagy plays a crucial role in the elimination of various intracellular bacteria from host cells. Activation of the mTORC1 pathway by Cif would inhibit autophagy and hence benefit the survival of the bacteria. Therefore, the effect of Cif on this pathway was determined by measuring the phosphorylation of the mTORC1 substrate p70 S6 kinase. As shown in Fig. 3A, there was no significant change in the phosphorylation levels of p70 S6K when Cif was transfected compared to cells transfected with empty vector. As expected, the mTORC1 inhibitor rapamycin inhibited p70 S6K phosphorylation. The result suggests that the mTOR pathway is not a target of Cif.

It has been reported that the MAPK pathways are common targets of many bacterial effectors (Mota and Cornelis, 2005; Bhavsar et al., 2007). Therefore, the effect of Cif on MAPK pathways was investigated. Interestingly, when Cif was transfected into cells, an increase in the phosphorylation levels of p38 MAPK and JNK1/2 was detected when compared to control cells (Fig. 3A). Similarly, Cif induced the phosphorylation of ERK1/2 MAPK, but notably to a much greater extent compared to p38 or JNK (Fig. 3B). Although the catalytically inactive Cif mutant exhibited a lower expression compared to wild-type Cif, as previously observed in Fig. 1B, the mutant Cif was without any effect on the phosphorylation level of ERK (Fig. 3B). This suggests that Cif-induced ERK activation is dependent on the catalytic activity of Cif. Thus, our results suggest that Cif upregulates the MAPK pathways with the most potent effect on ERK MAPK activation.

### Cif-dependent basal NF-κB activation is ERK MAPK dependent

**Fig. 4.**
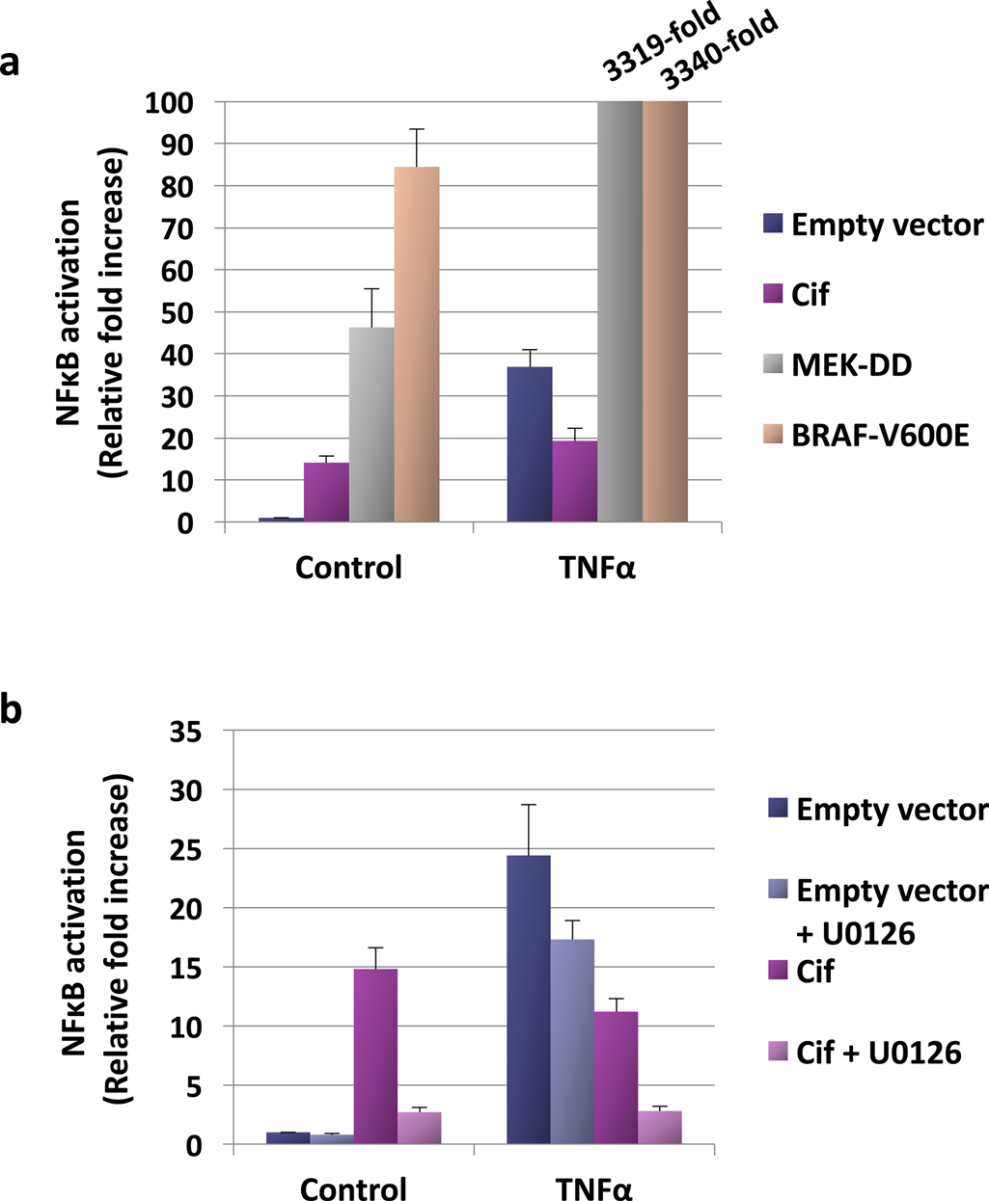
**Mechanism of Cif-dependent basal NFκB activation.** (A,B) Cells transfected with the indicated expression plasmids and an IL8 luciferase reporter plasmid were treated with 10 μM MEK inhibitor U0126 and stimulated with 30 ng/ml TNFα for 8 h, as described in the Materials and Methods. NF-κB activation was measured at the stipulated time and fold change was presented as mean±s.d. The results shown are representative of two independent experiments.

We next investigated the mechanism responsible for the basal NF-κB activation by Cif. Upon bacterial invasion, the MAPK-mediated inflammatory responses are often up-regulated as part of the host innate immune defence. In addition, Cif has been found in this study to induce activation of MAP kinases, in particular of ERK (Fig. 3). Therefore, we hypothesised that Cif stimulates basal NF-κB activity via ERK pathway activation. To test this, the effect of ERK pathway activation on NF-κB activity was first examined using the IL8 reporter system. The constitutive activators of the ERK pathway, MEK-DD and BRAF-V600E, were transfected into cells and the effect on NF-κB activation was measured. As shown in Fig. 4A, constitutive activation of the ERK pathway with either MEK-DD or BRAF-V600E resulted in a marked induction of NF-κB activity. We also noted that TNFα and the constitutively active ERK activators had a synergistic effect, causing an over 3000-fold induction of NF-κB activity. These results confirm that ERK activation is sufficient to activate NF-κB activity in HEK293T cells.

We next determined the effect of ERK pathway inhibition on NF-κB activity. Based on our hypothesis, it was predicted that inhibition of the ERK pathway would prevent the Cif-mediated increase in basal NF-κB activity. Indeed, as shown in Fig. 4B, when the ERK pathway was inhibited by treating cells with the MEK inhibitor U0126, Cif-induced basal NF-κB activation was largely blocked. Therefore, this result supports the hypothesis that Cif stimulates basal NF-κB activity via ERK pathway induction. We previously observed that Cif inhibits TNFα-induced NF-κB activation only partially (Fig. 2A). Hence, we also tested whether the lack of complete inhibition by Cif is due to simultaneous ERK pathway activation. As shown in Fig. 4B, when Cif-transfected cells were stimulated with TNFα and simultaneously treated with the MEK inhibitor, complete inhibition of NF-κB activity was observed. We therefore concluded that Cif inhibits TNFα-dependent NF-κB activation only partially because of a simultaneous Cif-mediated basal NF-κB activation.

### Effect of Cif on *B. thailandensis* intracellular replication

**Fig. 5.**
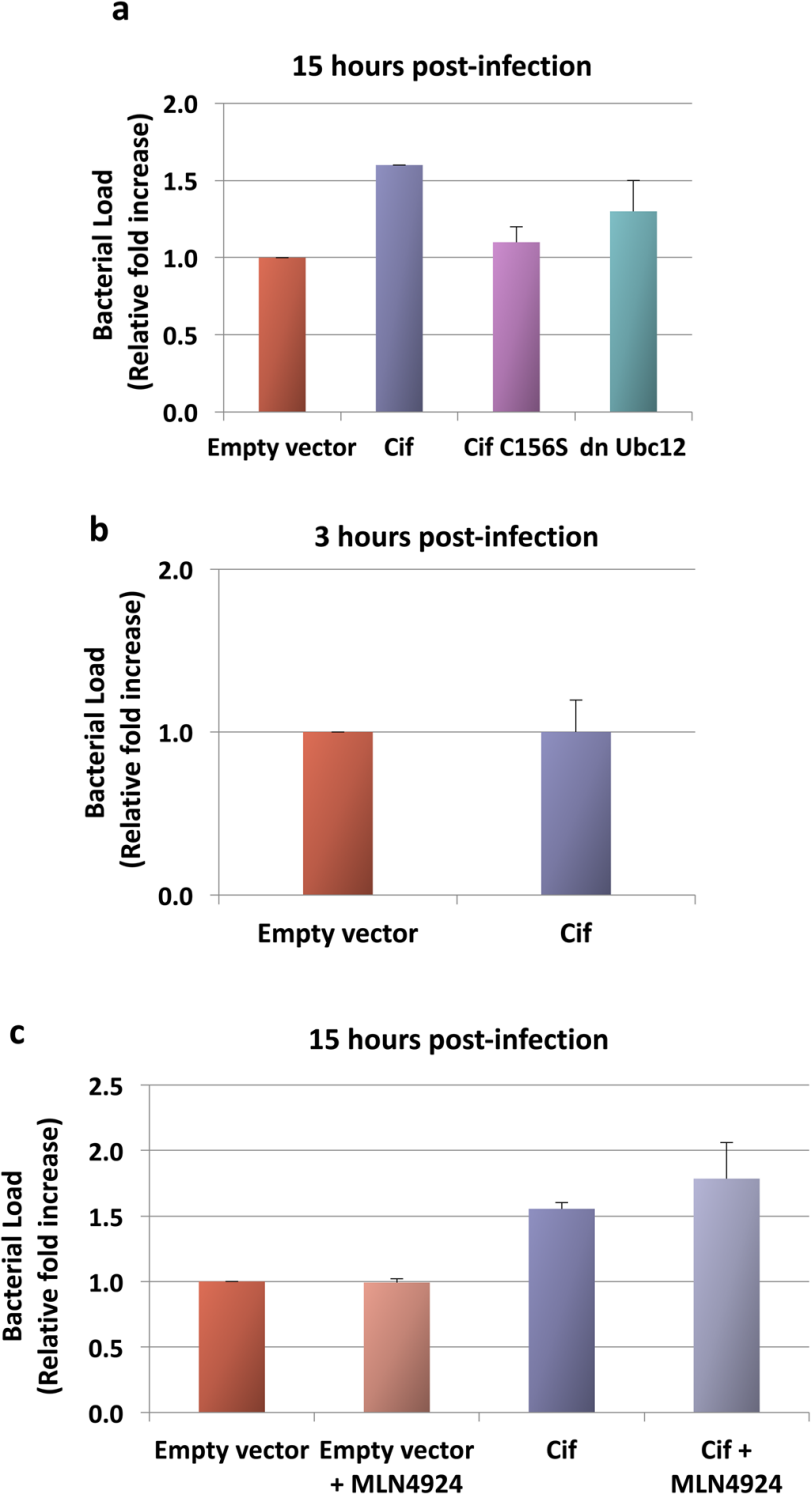
**Effect of Cif on *B. thailandensis* intracellular replication.** (A-C) Cells transfected with Cif, Cif C156S or dnUbc12 were pre-treated with 1 μM MLN4924 1 h prior to *B. thailandensis* infection at MOI 10:1. Bacterial load 15 h post-infection was assayed using colony-forming units on agar. (A) Fold change was presented as mean±s.e.m. of three independent experiments. (B,C) Fold change was presented as mean±s.d. of biological triplicates.

We then wanted to investigate whether Cif-dependent inhibition of CRLs and activation of ERK MAPK affects bacterial replication. To this end, HEK293T cells were transiently transfected with Cif followed by infection with *B. thailandensis*; a non-pathogenic surrogate of *B. pseudomallei*. We then assayed the intracellular replication of *B. thailandensis*, as described below, in the Materials and Methods. When cells were transfected with Cif, a 1.6-fold increase in *B. thailandensis* intracellular bacterial load was detected compared to the control cells transfected with empty vector (Fig. 5A). Although this effect is modest, it was robust and reproducible and, importantly, it provided a functional assay to study the cellular effects of Cif mechanistically.

Since Cif has been characterised as a deamidase, we next tested if the effect of Cif to promote *B. thailandensis* intracellular replication is dependent on its catalytic activity. To this end, cells were transfected with the catalytically inactive Cif C156S mutant, followed by a 15 h infection. As shown in Fig. 5A, at 15 h post-infection, no significant increase in bacterial load was observed with the catalytically inactive mutant compared to the control cells. This suggests that the proliferative effect of Cif on *B. thailandensis* intracellular replication is dependent on its catalytic activity.

It is possible that Cif does not directly increase *B. thailandensis* proliferation but instead causes enhanced bacterial internalisation. Therefore, we also tested whether Cif affects the rate of *B. thailandensis* internalisation by quantifying the intracellular bacterial loads 3 h post-infection. We observed no difference in the bacterial loads of Cif-transfected cells compared to control cells (Fig. 5B). This suggests that the bacterial growth promoting effect of Cif is independent of the rate of bacterial internalisation and supports the hypothesis that Cif has a direct effect on *B. thailandensis* intracellular replication.

We then tested whether the effect of Cif on *B. thailandensis* intracellular replication is due to inhibition of CRL activity. To this end, cells were transfected with a dominant negative Ubc12 (dnUbc12) plasmid. Ubc12 functions as a NEDD8 E2-conjugating enzyme; the dnUbc12 protein sequesters NEDD8, thus blocking CRL activity (Wada et al., 2000; Podust et al., 2000; Chew et al., 2007). Compared to transfection with Cif, transfection with dnUbc12 resulted in no significant increase in bacterial load. This suggests that the proliferative effect of Cif is not, or is only partially, dependent on CRL inhibition. We also used the NEDD8 E1-activating enzyme inhibitor MLN4924. When cells were treated with MLN4924, there was also no significant change in the bacterial load compared to untreated cells (Fig. 5C). It is possible that the lack of effect of MLN4924 was due to a simultaneous, CRL-independent inhibitory effect of the drug on bacterial replication; the drug may have exerted a direct inhibitory effect on *B. thailandensis*. To rule out this possibility, we treated Cif-transfected cells (in which CRL activity was already inhibited) with MLN4924. As shown in Fig. 5C, no decrease in bacterial replication was observed. This indicates that MLN4924 exerts no CRL-independent inhibitory effect on bacterial replication. Taken together, these results indicate that the effect of Cif in promoting bacterial intracellular replication is not, or is only partially, dependent on CRL inhibition.

### The effect of Cif to promote *B. thailandensis* intracellular replication is not due to ERK activation

**Fig. 6.**
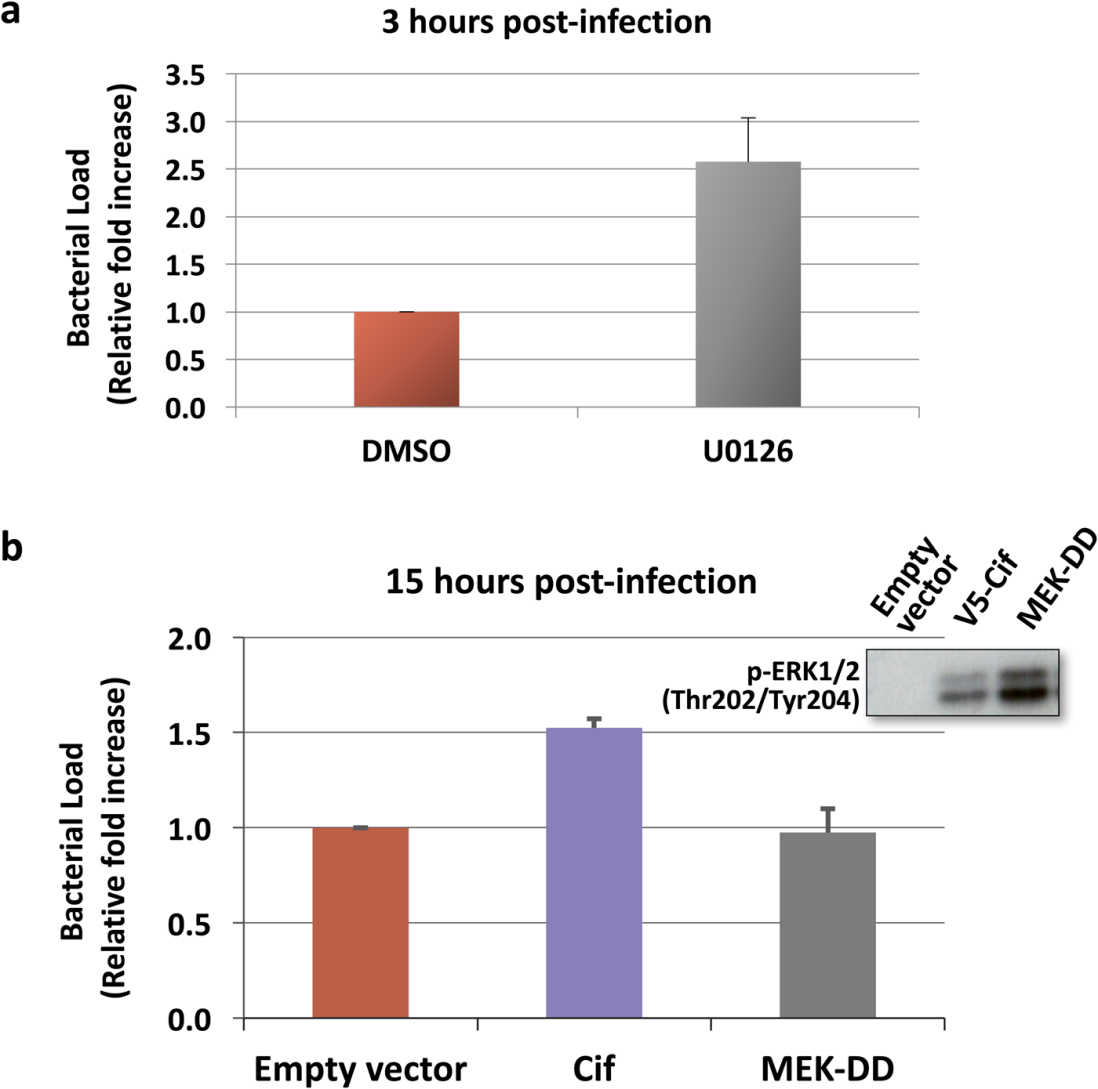
**The effect of Cif on *B. thailandensis* intracellular replication is not due to ERK MAPK activation.** (A) Cells were pre-treated with 10 μM MEK inhibitor U0126 for 2 h prior to *B. thailandensis* infection. Bacterial load 3 h post-infection was quantified. Fold change was presented as mean±s.d. of biological triplicates. (B) To measure the effect of ERK MAPK activation on *B. thailandensis* intracellular replication, cells were transfected with the constitutive activator of the ERK pathway, MEK-DD, for 2 days, and infected with *B. thailandensis*. Bacterial load 15 h post-infection was quantified. Fold change was presented as mean±s.e.m. of four independent experiments.

We next hypothesised that the ability of Cif to promote *B. thailandensis* intracellular replication is mediated through ERK activation. To test this hypothesis, we wanted to determine if blocking the ERK pathway with an ERK inhibitor would impair the effect of Cif to promote *B. thailandensis* replication. Given that p38 and JNK inhibitors have previously been reported to promote bacterial internalisation (Tang et al., 1998; Hu et al., 2006), we first tested the effect of the ERK pathway inhibitor U0126 on the internalisation of *B. thailandensis* in HEK293T cells. As shown in Fig. 6A, the ERK inhibitor also markedly increased the internalisation rate. Thus, this result precluded the use of the ERK pathway inhibitor in proliferation assays in Cif-transfected cells.

As an alternative approach, we determined if ERK activation was sufficient to increase bacterial load by using the constitutively active MEK-DD and BRAF-V600E plasmids. As shown in Fig. 6B (inset), the phosphorylation of ERK1/2 was markedly induced upon transfection of these plasmids. Of note, the effect of the constitutive activators on ERK activation was even greater compared to that of Cif. To determine if the bacterial growth promoting effect of Cif on *B. thailandensis* intracellular replication may be mediated via ERK pathway activation, the constitutive activators of the ERK pathway were transfected into cells and the bacterial loads were quantified at 15 h post-infection. As shown in Fig. 6B, there was no increase in *B. thailandensis* bacterial loads upon ERK pathway activation. In fact, bacterial replication was inhibited in MEK-DD and BRAF-V600E-transfected cells compared to cells transfected with empty vector. Taken together, these results indicate that ERK pathway activation by Cif is unlikely to be involved in promoting *B. thailandensis* intracellular replication. Hence, Cif activates *B. thailandensis* intracellular replication via a different mechanism.

## DISCUSSION

In this study, we aimed to characterise the cellular effects of Cif and identify targets of the Cif protein. We were specifically interested in determining whether inhibition of Cullin RING E3 ligases is the only cellular effect of Cif and whether CRL inhibition contributes to increased bacterial infection.

CRLs appear to be the major cellular target of Cif. Cif inhibits CRL function via deamidation of glutamine 40 in NEDD8 to glutamate. As shown in Fig. 1, upon transfection of Cif*_Bp_*, a very robust CRL inhibition can be observed, as indicated by a marked accumulation of CRL substrates to a degree that is even higher compared to treatment with the CRL (NEDD8 E1 inhibitor) inhibitor MLN4924. We also showed that Cif*_Yp_* has a similar CRL inhibitory effect. The actual mechanism through which Gln40 deamidation leads to CRL inhibition has been elucidated (Boh et al., 2011; Yu et al., 2015). Based on biochemical studies, we proposed that Cif-mediated deamidation of Gln40 interferes with the required conformational change in the CRL complex that is normally induced by NEDD8 (Boh et al., 2011). This mechanism has recently been confirmed in structural studies by (Yu et al., 2015).

By inhibiting CRL ubiquitin ligase activity, Cif causes the deregulation of a number of host cell functions. These include inhibition of cell cycle progression (via accumulation of cell cycle inhibitory proteins p21 and p27) as well as inhibition of the perforin-2- and NF-κB-dependent innate immune responses. However, our bacterial replication assay using *B. thailandensis* suggested that these CRL-dependent cellular effects of Cif are unlikely to be responsible for the Cif-dependent stimulation of *B. thailandensis* replication. Nonetheless, whether Cif-mediated CRL inhibition is of importance during *B. pseudomallei* *in vivo* infection remains to be determined.

We found that one additional cellular target of Cif is the ERK MAPK pathway. We have recently shown that activation of ERK by Cif is CRL independent (Ng et al., 2017). However, ERK activation was not involved in the Cif-mediated stimulation of *B. thailandensis* replication. Thus, there may be further, yet unidentified cellular targets of Cif.

We also found that Cif transfection at high levels had only a moderate effect on *B. thailandensis* intracellular replication. This suggests that at least in our experimental system, Cif plays only a minor role as a virulence factor. Of note, many pathogenic *B. pseudomallei* strains lack the Cif gene, further suggesting that Cif is not an essential virulence factor in *B. pseudomallei.* In contrast, Cif has been shown to be of pathological significance during *Y. pseudotuberculosis* infection in mice where Cif promotes pathogen survival (McCormack et al., 2015). Our results suggest that the differences in pathological significance are not due to a major divergence in the CRL inhibitory activity of the Cif homologues from *B. pseudomallei* and *Y. pseudotuberculosis*. However, the precise role of Cif in infection with *B. pseudomallei* and other Cif expressing pathogens remains to be further characterised.

In conclusion, both *B. pseudomallei* and *Y. pseudotuberculosis* exhibit marked CRL inhibitory activity. In addition, our results show that besides CRL inhibition, Cif has additional cellular effects, including ERK MAPK activation. In future work, it would be interesting to study CRL-independent functions of Cif in other pathogens where Cif may be of greater pathological significance compared to *B. pseudomallei*.

## MATERIALS AND METHODS

### Cell culture, drug treatment and transfection

HEK293T cells (American Type Culture Collection) were cultured in Dulbecco's modified eagle medium (DMEM) (Invitrogen), supplemented with 10% (vol/vol) heat-inactivated fetal bovine serum (HyClone), 4 mM L-glutamine (Invitrogen), 50 U/ml penicillin and 100 mg/ml streptomycin (Invitrogen) in a humidified 37°C, 5% CO_2_ tissue culture incubator. For drug treatment, the NEDD8 E1 ubiquitin activating enzyme (NAE1) inhibitor MLN4924 was used at 1 μM, a concentration that was found to be required for complete inhibition of NAE1 in lymphoma cells and that is commonly used in cell-based experiments (Milhollen et al., 2010; Soucy et al., 2009). The mTORC1 inhibitor rapamycin was used at 20 nM and the MEK inhibitor U0126 at 10 μM. These concentrations are commonly used in mammalian cell-based studies. In particular, Favata et al. (1998) originally measured IC50 values for the MEK inhibitor U0126 of 1 to 2 μM in several cell-based assays. Hence, our concentration of 10 μM (5 to 10 times the IC50) appears appropriate and is widely used in the literature. Transient transfections were performed using Genejuice transfection reagent (Novagen) in accordance with the manufacturer's directions for sub-confluent cells.

### Plasmid constructs

The FLAG- or V5-Cif pcDNA3.1 plasmids bearing an N-terminal 2xFLAG or V5 epitope tag sequence used were previously described (Boh et al., 2011). The Cif mutant (V5-Cif C156S) was obtained via site-directed mutagenesis using forward primer 5′-GAT GAC GCC CGT GTC CGG ACT TTC GGC CA-3′ and reverse primer 5′-TGG CCG AAA GTC CGG ACA CGG GCG TCA TC-3′, and cloned into pcDNA3.1 with 5′ KpnI and 3′ XbaI. The Cif*_Yp_* sequence optimised for *Homo sapiens* was synthesised by ShineGene Molecular Biotech Inc. and cloned into the N-terminal V5 epitope tag-bearing plasmid backbone using 5′ KpnI and 3′ XhoI. The NF-κB reporter contained the 2415/293 fragment of the human IL8 promoter cloned into the KpnI and HindIII restriction sites of the pGL2 plasmid (Promega).

### Immunoblotting

Cells were washed with ice-cold Phosphate-Buffered Saline (PBS) and then lysed in Triton X-100-containing lysis buffer. The composition of the lysis buffer was as follows: 25 mM Tris-HCl (pH 7.5), 100 mM NaCl, 2.5 mM EDTA, 2.5 mM EGTA, 20 mM NaF, 1 mM Na_3_VO_4_, 20 mM Sodium β-Glycerophosphate, 10 mM Sodium Pyrophosphate, 0.5% Triton X-100, Roche protease inhibitor cocktail and 0.1% β-Mercaptoethanol. Lysates were pre-cleared by centrifugation before use for Western blotting. Equal amounts of protein were loaded for Western blot analysis. All the following monoclonal antibodies used were obtained from Cell Signaling Technology: anti-phospho-p70S6K (Thr389) (#9234), anti-phospho-p38 (Thr180/Tyr182) (#9215), anti-phospho-JNK1/2 (Thr183/Tyr185) (#4668), anti-phospho-ERK1/2 (Thr202/Tyr204) (#9101), anti-phospho-IκBα (Ser32) (#2859), and anti-IκBα (#9242), except for anti-p27 (BD Biosciences, #610241), anti-HIF-1α (BD Biosciences, #610959), anti-α-tubulin (Molecular Probes, #236-10501) and anti-V5 (Serotec, #MCA1360). All the antibodies were used at dilutions of between 1:10,000 and 1:5000. The Western blots shown are representative of at least two independent experiments.

### Intracellular survival and replication of *B. thailandensis*

Cells were cultured in 12-well plates and transfected with 0.8 μg empty vector pcDNA3.1, V5-Cif-pcDNA3.1, V5-Cif C156S-pcDNA3.1, dnUBC12-HA C111S-pcDNA3 (Chew et al., 2007), pBabe-Puro-MEK-DD or pBabe-Puro-BRAF-V600E [Addgene plasmids #15268 and #15269, a gift from William Hahn (Boehm et al., 2007)]. The culture medium was replaced the following day with antibiotic-free DMEM. Cells were infected at a multiplicity of infection (MOI) of 10:1 with mid-log-phase bacteria. Mid-log-phase *B. thailandensis* culture was prepared by inoculating 500 μl of overnight culture into 5 ml Luria-Bertani (LB) broth and incubated for 1 h with constant agitation. Infected cells were centrifuged at 600 ***g*** for 10 min to allow for host cell contact and then incubated at 37°C. At 2 h post-infection, 250 μg/ml kanamycin and 10 μg/ml imipenem were added to kill off extracellular bacteria. Infected cells were washed once with PBS and lysed at 15 h or 3 h post-infection with 1 ml 0.1% Triton X-100. Serial dilutions of the lysates were plated onto TSA agar (six replicates) and incubated at 37°C overnight. Colony forming units were then used to calculate bacterial loads. For the inhibitor studies, the same transfection and infection procedures were adopted, except that transfected cells were pre-treated with 1 μM MLN4924 for 1 h, or 10 μM U0126 for 2 h, after replacement with antibiotic-free medium prior to infection.

### IL8 luciferase reporter assay for detection of NFκB activation

Cells were cultured in 12-well plates and co-transfected with 0.1 μg IL8 luciferase reporter plasmid and 0.4 μg empty vector pcDNA3.1, V5-Cif-pcDNA3.1 or V5-Cif C156S-pcDNA3.1 for 24 h. Cells were then treated with 1 μM MLN4924 in duplicates for 8 h where indicated. To stimulate the NFκB pathway, cells were co-treated with 30 ng/ml of TNFα (Miltenyi Biotec) for 8 h. To examine NFκB activation by *B. thailandensis*, cells were infected with mid-log-phase bacteria 1 h post-drug treatment at an MOI of 10:1 in antibiotic-free medium for 15 h. For the infected cells, at 2 h post-infection, 250 μg/ml kanamycin and 10 μg/ml imipenem were added to kill off extracellular bacteria. Cells were lysed in Steady-Glo^®^ Luciferase Assay System (Promega) and luciferase expression was determined using a 20/20*^n^* Luminometer (Turner Biosystems). For MAPK-mediated inflammatory response studies, cells were co-transfected with IL8 luciferase reporter plasmid and empty vector pcDNA3.1, V5-Cif-pcDNA3.1, pBabe-Puro-MEK-DD or pBabe-Puro-BRAF-V600E plasmid. Transfected cells were treated with 10 μM U0126 and stimulated with 30 ng/ml TNFα for 8 h before NFκB activation was assayed.
